# Preparation of Glycerol-Enriched Yeast Culture and Its Effect on Blood Metabolites and Ruminal Fermentation in Goats

**DOI:** 10.1371/journal.pone.0094410

**Published:** 2014-04-07

**Authors:** Gengping Ye, Yongxing Zhu, Jin Liu, Xingxiang Chen, Kehe Huang

**Affiliations:** Institute of Nutritional and Metabolic Disorders in Domestic Animals and Fowls, Nanjing Agricultural University, Nanjing, China; University of Nottingham, United Kingdom

## Abstract

The aim of this study was to isolate a glycerol-producing yeast strain from nature to prepare glycerol-enriched yeast culture (GY), and preliminarily evaluate the effects of GY on blood metabolites and ruminal fermentation in goats. During the trial, six isolates were isolated from unprocessed honey, and only two isolates with higher glycerol yield were identified by analysis of 26S ribosomal DNA sequences. One of the two isolates was identified as *Saccharomyces cerevisiae*, a direct-fed microbe permitted by the FDA. This isolate was used to prepare GY. The fermentation parameters were optimized through single-factor and orthogonal design methods to maximize the glycerol yield and biomass. The final GY contained 38.7±0.6 g/L glycerol and 12.6±0.5 g/L biomass. In vivo, eight castrated male goats with ruminal fistula were used in a replicated 4×4 Latin square experiment with four consecutive periods of 15 d. Treatments were as follows: control, LGY, MGY, and HGY with 0, 100, 200, and 300 mL GY per goat per day, respectively. The GY was added in two equal portions at 08∶00 and 17∶00 through ruminal fistula. Samples of blood and ruminal fluid were collected on the last one and two days of each period, respectively. Results showed that the plasma concentrations of triglyceride and total cholesterol were not affected by the supplemented GY. Compared with the control, goats supplemented with MGY and HGY had significantly higher (*P*<0.05) concentrations of plasma glucose and total protein, ruminal volatile fatty acid and molar proportion of propionate, and significantly lower (*P*<0.05) ruminal pH and ammonia nitrogen. These parameters changed linearly with increasing GY supplementation level (*P*<0.05). In conclusion, GY has great potential to be developed as a feed additive with dual effects of glycerol and yeast for ruminants.

## Introduction

During the perinatal period, satisfying the nutritional requirements of ruminants is challenging. Dry matter intake may decrease by as much as 30% on the week before calving [1], and often continues to be insufficient to meet the increasing energy demands of lactation in the first 5 weeks after calving. Thus, ruminants, especially cows and ewes, frequently enter a state of negative energy balance, in which energy output in the form of milk exceeds energy input in the form of feed [2]. To compensate for this energy deficiency, ruminants increase the mobilization of body fat. Consequently, increased lipolysis causes an elevation of nonesterified fatty acids and excessive accumulation of ketobodies in blood, which will result in many energy metabolic diseases that can seriously affect the health and performance of ruminants.

Because of our inability to overcome the intake depression observed around calving, and ruminal metabolism characteristic that glucose is directly broken down by ruminal microorganisms but not absorbed across the ruminal wall, producers often feed glucogenic precursors to ruminants to provide energy for the body via gluconeogenesis pathway. Glycerol is an important structural component of triglycerides and phospholipids, and frequently used as a gluconeogenic substrate supplemented in the diets of ruminants because of its well-established glucogenic properties [3]. After oral administration, glycerol is readily absorbed through the ruminal wall and converted to glucose via the gluconeogenesis pathway in the liver [4], or fermented to propionate in rumen and acts as a precursor for hepatic glucose synthesis [5]. Previous study reported that oral administration of glycerol can rapidly increase blood glucose and reduce blood ketones in ewes with pregnancy toxaemia, in comparison with intravenous glucose injections [6]. Moreover, early lactation cows supplemented with glycerol possess higher plasma glucose concentrations compared with control cows [7], [8]. The plasma concentration of glucose linearly increases as the supplementation level of glycerol increases [8].

With the recent expansion of the biofuel industry, glycerol has been excessively produced as a byproduct of biodiesel production, making it a more attractive energy supplement for ruminants. However, the glycerol originating from biodiesel production contains several deleterious impurities (e.g., methanol) [2] that are generated during glycerol synthesis or extraction. Even 85% glycerol still contains 0.04% (400 ppm) methanol, which is significantly higher than the 150 ppm tolerable limit set by the FDA for animals [9]. In addition, high-purity glycerol is costly in the present situation. Therefore, in the present study, glycerol was produced by fermentation with a direct-fed microbe (yeast) permitted by the FDA, which has been proven capable of improving the diet digestibility, ruminal fermentation, and performance of sheep [10]–[12] and cows [13], [14]. The final broth, glycerol-enriched yeast culture (GY), was first proposed to be added to ruminant diets as a new feed additive. The GY not only avoids the generation of deleterious impurities and the extraction of glycerol, but also combines the beneficial effects of glycerol and yeast.

This study aims to isolate a glycerol-producing yeast strain from nature to prepare GY, and preliminarily evaluate the effects of GY on blood metabolites and rumen fermentation in goats.

## Materials and Methods

### Isolation

The isolates were derived from unprocessed honey bought from an apiary in Nanjing, China. One gram sample of honey was dissolved in 100 mL of sterilized physiological saline, serially diluted with sterilized physiological saline to 10^−10^ and suspended. Then, 100 μL suspension at each dilution ratios were respectively cultivated on plates with malt extract medium (10 g/L glucose, 15 g/L malt extract, 3 g/L yeast extract, 20 g/L agar, and pH 5.4, sterilized at 121°C for 15 min) supplemented with 100 mg/mL ampicillin sodium. The plates were incubated at 28°C in a homothermal incubator until a single colony could be observed. White/cream or turbid yeast-like colonies that matched the morphological properties indicated in the standard taxonomic key were obtained [15], and then transferred to a yeast extract peptone dextrose medium (20 g/L glucose, 10 g/L yeast extract, 10 g/L peptone, and 2 g/L agar, sterilized at 121°C for 15 min) for purification. All purified isolates were investigated for their ability to produce glycerol by fermentation. The isolates with high glycerol yield were selected for identification.

### Identification of Yeast Strains by 26S Ribosomal DNA D1/D2 Domain Sequences

The DNA of the selected isolates was extracted by single-tube LiOAc-SDS lysis [16], and fragments containing the D1/D2 region approximately 550 bp to 650 bp at the 5′ end of the 26S rDNA were amplified using primers: NL-1 (5′-GCATATCAATAAGCGGAGGAAAAG-3′) and NL-4 (5′-GGTCCGTGTTTCAAGACGG-3′). Polymerase chain reactions (PCR) was performed in a 50 μL reaction mixture containing 10 mM Tris-HCl, 50 mM KCl, 1.5 mM MgCl_2_, 0.2 mM of each dNTP, 1.25 IU of Taq polymerase, 0.2 mM of each primer and 1 μL of DNA template. The PCR amplification was conducted under the following conditions: initial denaturation at 95°C for 3 min; 34 cycles of 95°C for 15 s, 59°C for 1 min, and 72°C for 2 min; final extension at 72°C for 10 min; and holding at 4°C [17]. The amplified products were purified and sequenced. The obtained sequence data were submitted to the GenBank database and compared with all known yeast species in the database. Among these strains, the one identified as a direct-fed microbe permitted by the FDA was selected for the next experiments.

### Fermentation and Optimization of GY Fermentation Parameters

A single fresh colony of the identified yeast strain was inoculated into 50 mL of sterilized seed medium (20 g/L glucose, 10 g/L yeast extract, and 20 g/L peptone) in a 250 mL Erlenmeyer flask and then incubated in a rotary shaker at 150 rpm for 24 h at 28°C. Then, a 10 mL seed medium was inoculated into a 90 mL sterilized basal fermentation medium (200 g/L glucose, 5 g/L yeast extract, 5 g/L corn steep liquor powder (CSLP), and 0.1 g/L MgSO_4_·7H_2_O) in a 500 mL Erlenmeyer flask and then incubated in a rotary shaker at 180 rpm for 48 h at 30°C. Finally, the glycerol yield and biomass (dry cell weight, DCW) in the final broth were determined.

The effects of different medium components and fermentation conditions were investigated in 500 mL Erlenmeyer flasks and a 30 L fermenter to maximize glycerol yield and DCW. Glycerol yield was the main determinant to select each factor and level. The former optimal factor and level were used as the fermentation parameter to optimize the latter parameter. Optimization parameters were as follows: glucose as carbon source at different final concentrations (100 g/L to 500 g/L in 50 g/L increments); yeast extract, peptone, urea, and NH_4_Cl as nitrogen sources at 5 g/L followed by optimal nitrogen source at different final concentrations (1, 3, 5, 7, and 9 g/L); different phosphorus sources (K_2_HPO_4_, KH_2_PO_4_, and CSLP) at 2 g/L followed by optimal phosphorus source at different final concentrations (1, 3, 5, 7, and 9 g/L). Different concentrations of NaCl (0 g/L to 80 g/L in 10 g/L increments) were added to the basal medium to obtain the desired hyperosmotic environment. In addition, the effects of inoculum size (3%, 5%, 10%, 15%, and 20%) and fermentation time (0 h to 120 h in 12 h increments) on glycerol yield and DCW were also investigated with single-factor experiments. Unlike the above optimization performed in 500 mL Erlenmeyer flasks, temperature (28°C, 30°C, 32°C, and 34°C), constant pH (4, 5, 6, and 7), and dissolved oxygen (DO, 40%, 60%, 80%, and 100%) were optimized by orthogonal test L_16_ (4^5^) in a 30 L fermenter containing 10 L of basal fermentation broth because these conditions are difficult to control in Erlenmeyer flasks.

### Animal and Experimental Design

All experimental procedures involving animals were approved by the Animal Care and Use Committee of Nanjing Agricultural University, and the animal ethical number is SYXK (Su) 2011-0036.

Eight castrated male goats (2 to 3 years old) with ruminal fistula were selected and used in a replicated 4×4 Latin square experiment with four consecutive periods of 15 d. The goats were housed in individual pens (2 m×3 m) and fed twice daily at 08∶00 and 17∶00 with fresh water available throughout the experimental period. The treatments were as follows: control, LGY, MGY, and HGY with 0, 100, 200, and 300 mL GY per goat per day, respectively. GY was added in two equal portions at 08∶00 and 17∶00 through the ruminal fistula. Each goat received a basal diet containing 90 g concentrate and 180 g alfalfa hay per time. The concentrate contained 65% corn, 25% soybean meal, and 10% wheat bran, whereas the alfalfa hay contained 90.54% dry matter, 17.52% crude protein, 2.62% crude fat, 29.05% crude fiber, 1.64% Ca, and 0.36% P.

Blood samples were collected approximately 2 h after morning feeding on day 15 of every period from the jugular vein into 10 mL vacuum tubes containing Na-EDTA as anticoagulant. Plasma was immediately separated from whole blood by centrifuging at 1000×g for 10 min at 4°C. All plasma samples were stored at −20°C until determination of glucose, total protein, urea N, cholesterol and triglyceride.

Ruminal pH and fermentation characteristics were measured on the last two consecutive days (days 14 and 15) of each period. At 0, 3, 6, and 9 h after the morning feeding, ruminal fluid (20 mL) was collected through the ruminal fistula using a tube fitted with a suction strainer. The collected fluid was filtered through four layers of cheesecloth and then immediately analyzed for pH using a digital pH meter (pHS-3C, Tian Da instrument Co., Ltd., Shanghai, China). Following pH determination, 7.5 mL of ruminal fluid was added to 2.5 mL of 25% (w/v) metaphosphoric acid and stored at −20°C until determination of volatile fatty acid (VFA) and ammonia nitrogen (NH_3_N) levels.

### Analytical Methods

Microorganism growth was monitored by DCW and viable count. The final broth (5 mL) was sampled and centrifuged at 2770×g for 10 min and the sediment was washed twice with distilled water and dried at 85°C to constant weight to determine DCW. Viable count was detected by the plate count method [18]. Glycerol was measured directly by the colorimetric method [19].

The plasma concentrations of glucose, total protein, urea N, cholesterol, and triglyceride were determined using an automatic biochemical analyzer (Mindray BS-300, Mindray Medical International Limited, Shenzhen, China).

Ruminal fluid was thawed and centrifuged at 2000×g for 15 min at 4°C. The NH_3_N content was analyzed using a modified colorimetric method [20]. The VFA in the ruminal fluid was extracted with chloroform. Crotonic acid served as an internal standard. Then, 1 μL of the sample was injected for composition analysis using a gas chromatograph (Agilent 7890A, Agilent Technologies, Inc., Santa Clara, CA, USA) equipped with a DB-FFAP capillary column, 30 m×0.32 cm×50 μm (Agilent J&W Advanced Capillary GC Columns, Netherlands) and a flame ionization detector. The temperature of oven, injector, and detector were set at 150°C, 180°C, and 180°C, respectively. The split ratio was 20∶1. Helium was used as the carrier gas. The flow rates of helium, air, and H_2_ were set at 30, 450, and 40 mL/min, respectively.

### Data Analysis

Data of GY preparation were analyzed with SPSS 18.0 software (SPSS, Inc., Chicago, IL, USA). Differences among means were assessed by Duncan's multiple range tests of one-way analysis of variance (ANOVA). Data of blood metabolites were analyzed using the mixed model procedure of SAS 9.2 to account for the effects of period, animal and treatment. The treatment was considered as a fixed effect; period and animal were considered as random effects. However, data of ruminal fermentation was summarized by sampling time and then analyzed using the same mixed model but with time included as a repeated measure using compound symmetry. Linear and quadratic orthogonal contrasts were tested using the CONTRAST statement of SAS with coefficients estimated based on the GY application quantity. Values of GY preparation were presented as means ± standard deviation, and values of in vivo experiment were presented as least square means ± associated standard errors. Statistical significance was considered at *P*<0.05 and a tendency to significance was considered at 0.05≤*P*<0.10.

## Results

### GY Preparation

#### Isolation and Identification

**Figure 1:**
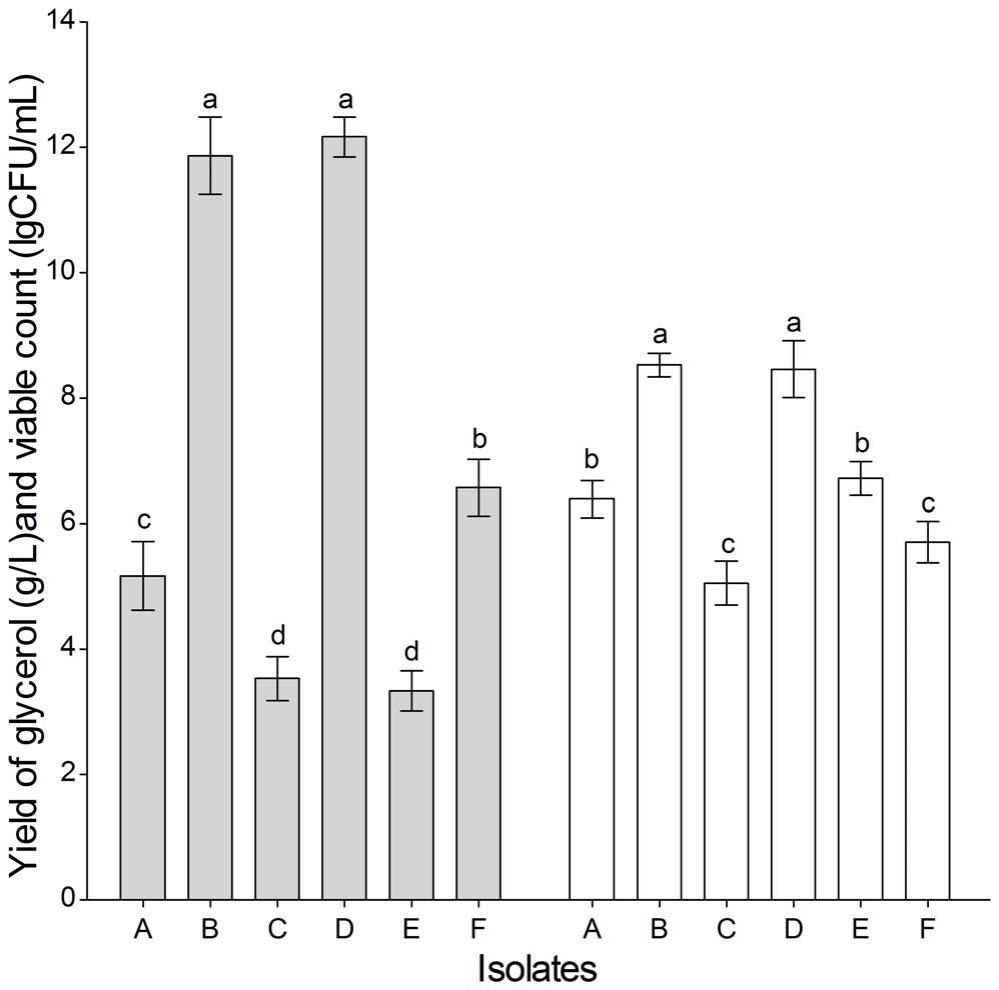
Fermentation results of six isolates. Bars with different letters are statistically significantly different (*P*<0.05) from each other by one-way ANOVA followed by Duncan's multiple range tests. Bars sharing a common letter have no significant differences (*P*>0.05). Symbols: ▪ glycerol yield, □ viable count.

In this study, six isolates were isolated from unprocessed honey. The glycerol yields and viable counts of the isolates are presented in Figure 1. Strains B and D had significantly higher (P<0.05) glycerol yields and viable counts compared with the other strains.

The 26S ribosomal DNA D1/D2 domain sequences of strains B and D were 572 and 581 bp respectively, and they had high homology with *Saccharomyces cerevisiae* and *Zygosaccharomyces rouxii* in the GenBank database (accession numbers: JF682845 and JN981152, respectively). The homologies of both strains B and D reached 99%. Strain B (*S. cerevisiae*), a direct-fed microbe permitted by the FDA, was selected to prepare GY in this study.

#### Optimization of Fermentation Medium Components

**Figure 2:**
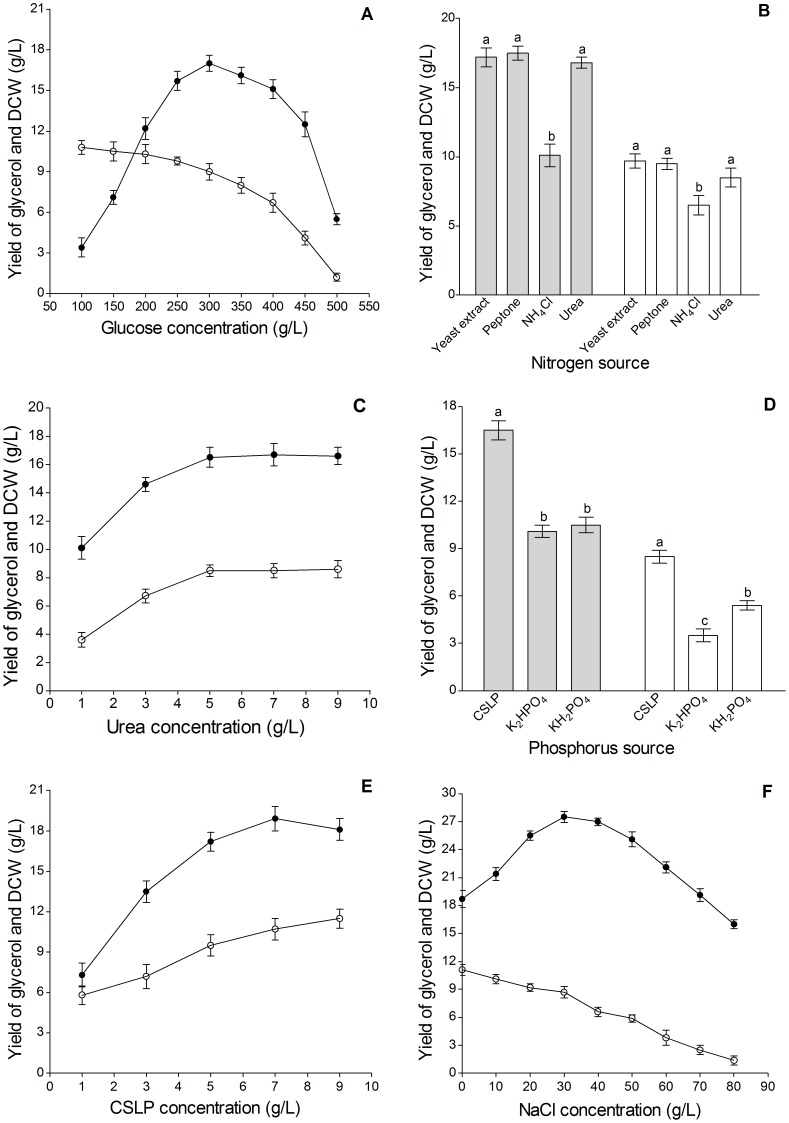
Effects of different medium components on glycerol yield and dry cell weight (DCW). Different glucose concentrations (A), nitrogen sources (B), urea concentrations (C), phosphorus sources (D), CSLP concentrations (E) and NaCl concentrations (F) were optimized to maximize the glycerol yield and DCW. Bars with different letters are statistically significantly different (*P*<0.05) from each other by one-way ANOVA followed by Duncan's multiple range tests. Bars sharing a common letter have no significant differences (*P*>0.05). Symbols: • or ▪ glycerol yield, ○ or □ DCW.

The effects of fermentation medium components on glycerol yield and DCW are presented in Figure 2. As shown in Figure 2A, the maximum glycerol yield of 17.1±0.6 g/L was achieved when the initial glucose concentration was 300 g/L. However, DCW decreased from 10.8±0.5 g/L to 1.2±0.1 g/L when the glucose concentration increased from 100 g/L to 500 g/L. Thus, 300 g/L glucose was used for the following experiments.

Yeast extract, peptone, and urea as nitrogen sources produced higher glycerol yields compared with NH_4_Cl (*P*<0.05, Figure 2B). Similar impact of different nitrogen sources on the DCW was observed. Finally, urea was selected as the optimal nitrogen source considering feedstock cost and source. In Figure 2C, glycerol yield increased to 16.5±0.7 g/L when the urea concentration increased to 5 g/L and remained stable afterward. Similar impact of different urea concentrations on the DCW was observed. Therefore, 5 g/L urea was selected as the optimal concentration.

In Figure 2D, CSLP as phosphorus source produced higher glycerol yield and DCW compared with K_2_HPO_4_ and KH_2_PO_4_ (*P*<0.05). As shown in Figure 2E, glycerol yield peaked at 18.7±0.7 g/L when the CSLP concentration was 7 g/L. The DCW increased with increasing CSLP concentration and reached 11.5±0.5 g/L when the CSLP concentration was 9 g/L. The optimal CSLP concentration was 7 g/L.

As shown in Figure 2F, the maximum glycerol yield of 27.5±0.6 g/L was reached when the NaCl concentration was 30 g/L. DCW decreased from 11.1±0.6 to 1.4±0.2 g/L when the NaCl concentration increased from 0 g/L to 80 g/L. Thus, 30 g/L NaCl was the optimal concentration.

To sum up, the optimal fermentation medium components were as follows: 300 g/L glucose, 5 g/L urea, 7 g/L CSLP, and 30 g/L NaCl.

#### Optimization of Fermentation Conditions

**Figure 3:**
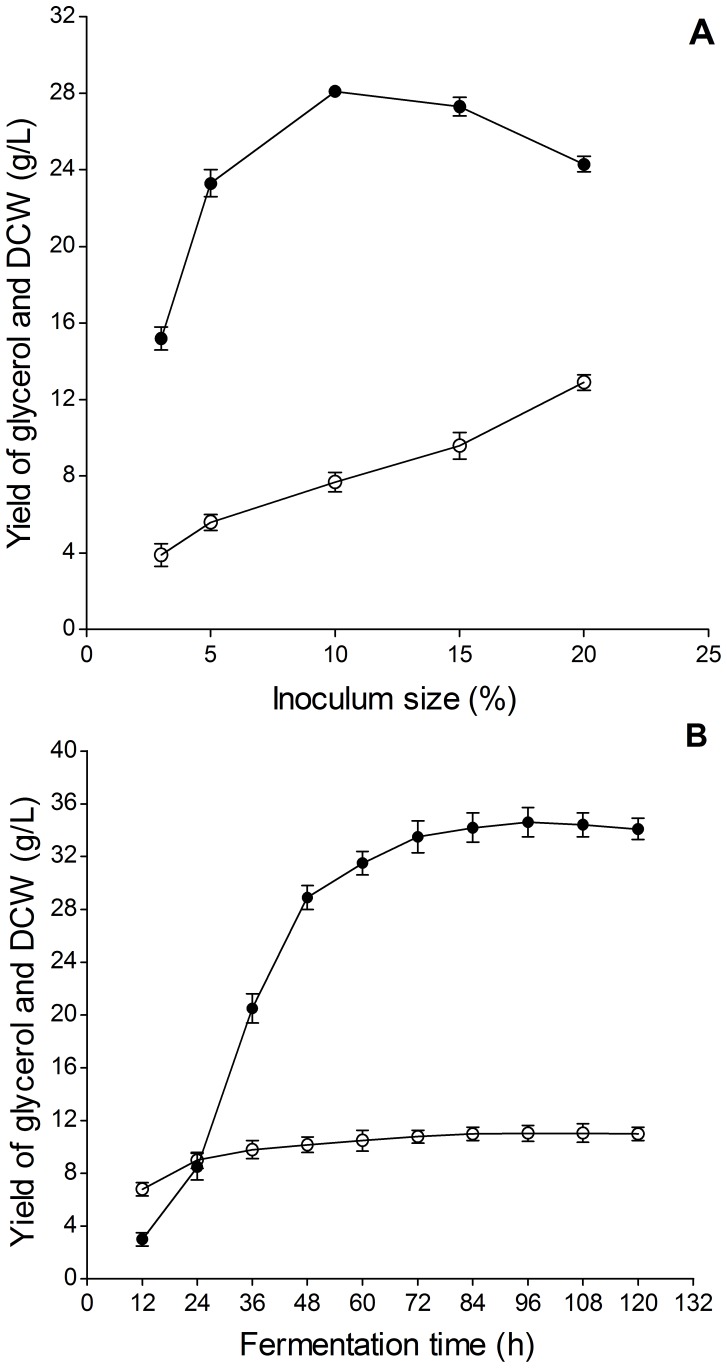
Effects of different inoculation sizes (A) and fermentation times (B) on glycerol yield and dry cell weight (DCW). Symbols: • glycerol yield, ○ DCW.

The effects of fermentation conditions on glycerol yield and DCW are shown in Figure 3. As depicted in Figure 3A, the optimal glycerol yield of 28.1±0.3 g/L was obtained at 10% inoculum size. However, DCW increased with increasing inoculum size and then peaked at 12.9±0.4 g/L at 20% inoculum size. The inoculum size at 10% was selected for the next experiments.

As shown in Figure 3B, glycerol yield increased substantially to 28.9±1.3 g/L before 48 h. Thereafter, it maintained a slowly upward trend until the peak at 96 h but only increased by 5.7 g/L within 2 d. DCW increased quickly before 24 h and reached 9.0±0.6 g/L at 24 h. Therefore, 48 h was chosen as the optimal fermentation time.

The factors and levels of fermentation conditions for orthogonal test and the analysis of L_16_ (4^5^) test results are shown in Tables 1 and 2, respectively. The effect of fermentation conditions on glycerol yield and DCW decreased in the order A>C>B according to the R_1_ and R_2_ values. According to the means of glycerol yields for each factor at different levels, the maximum yield of glycerol was obtained when the temperature, constant pH, and DO were 30°C, 5, and 80%, respectively. Under these fermentation condition, glycerol yield, DCW, and yeast viable count reached 38.7±0.6 g/L, 12.6±0.5 g/L, and 9.4±0.3 lg CFU/mL, respectively.

**Table 1:** Factors and levels of orthogonal test L_16_ (4^5^) for optimizing fermentation conditions.

	*Factors*		
Levels	A(T^1^, °C)	B(pH)	C(DO^2^,%)
1	28	4	40
2	30	5	60
3	32	6	80
4	34	7	100

1 Temperature.

2 Dissolved oxygen.

**Table 2:** Results of orthogonal test L_16_ (4^5^) and analysis.

No.	A(T^1^)	B(pH)	C(DO^2^)	Glycerol yield, g/L	DCW^3^, g/L
1	1	1	1	32.0	10.5
2	1	2	3	33.8	11
3	1	3	4	30.3	10.2
4	1	4	2	29.8	9.3
5	2	1	2	34.4	9.5
6	2	2	4	37.6	13.2
7	2	3	3	35.7	11.4
8	2	4	1	30.1	7.3
9	3	1	3	36.1	10.5
10	3	2	1	33.0	7.9
11	3	3	2	32.1	9.3
12	3	4	4	33.9	8.7
13	4	1	4	32.6	8.9
14	4	2	2	31.1	9.2
15	4	3	1	27.2	5.1
16	4	4	3	30.5	6.3
*K_11_* a	31.5	33.4	30.6		
*K_12_* a	34.5	33.9	31.9		
*K_13_* a	33.4	31.3	34.0		
*K_14_* a	30.4	31.1	33.6		
R_1_^b^	4.1	2.8	3.0		
*K_21_* c	10.1	9.9	7.7		
*K_22_* c	10.4	10.3	9.3		
*K_23_* c	9.1	9.0	9.8		
*K_24_* c	7.4	7.9	10.3		
R_2_^d^	3.0	2.4	2.6		

1 Temperature.

2 Dissolved oxygen.

3 Dry cell weight.

a and ^b^ were the means and ranges of glycerol yields for each factor at different levels, respectively.

c and ^d^ were the means and ranges of DCW for each factor at different levels, respectively.

### In Vivo Results

#### Effect of GY on Blood Metabolites in Goats

The concentrations of plasma glucose, total protein, urea N, total cholesterol, and triglyceride are presented in Table 3. The concentrations of plasma glucose and total protein on day 15 were both higher in the MGY and HGY groups than in the control group (P<0.05). Moreover, the concentrations of plasma glucose and total protein increased linearly with increasing GY supplementation level (P<0.05). However, the treatments did not affect the concentrations of plasma urea N, total cholesterol, and triglyceride.

**Table 3:** The plasma concentrations of glucose, total protein, urea N, total cholesterol, and triglyceride for goats supplemented with control, LGY, MGY and HGY^1^.

	*Treatments*					*Contrast, P*		
Item	Control	LGY	MGY	HGY	*SE*	Treatment	Linear	Quadratic
Glucose, mmol·L^−1^	2.82^b^	3.01^ab^	3.31^a^	3.33^a^	0.10	<0.05	<0.05	0.38
Total protein, g·L^−1^	60.88^b^	64.85^ab^	66.95^a^	66.42^a^	1.06	<0.05	<0.05	0.11
Urea N, mmol·L^−1^	4.65	4.60	4.71	4.57	0.05	0.20	0.53	0.41
Total cholesterol, mmol·L^−1^	2.52	2.48	2.46	2.50	0.02	0.17	0.39	0.05
Triglyceride, mmol·L^−1^	0.15	0.13	0.11	0.13	0.01	0.23	0.19	0.16

1 LGY, MGY and HGY  =  glycerol-enriched yeast culture at 100, 200 and 300 mL per goat per day, respectively.

ab Means within a row with different superscripts differ (*P*<0.05).

#### Effect of GY on Ruminal Fermentation in Goats

The ruminal fermentation characteristics of goats are shown in Table 4. Compared with the control group, the MGY and HGY groups had lower ruminal pH and NH_3_N but higher total VFA (*P*<0.05). These parameters changed linearly with increasing GY supplementation level (*P*<0.05). Molar proportion of propionate was significantly affected by HGY (*P*<0.05) and increased linearly with increasing GY supplementation level (*P*<0.05). Increased GY supplementation level did not affect the molar proportion of acetate and butyrate but linearly decreased the ratio of acetate to propionate (A: P) (*P* = 0.05).

**Table 4:** The ruminal pH, NH_3_N, and volatile fatty acid proportions for goats supplemented with control, LGY, MGY and HGY^1^.

	*Treatments*					*Contrast, P*		
Item	Control	LGY	MGY	HGY	*SE*	Treatment	Linear	Quadratic
pH	6.67^a^	6.63^a^	6.58^b^	6.52^c^	0.02	<0.05	<0.05	0.63
NH_3_N, mg/dL	10.28^a^	10.10^ab^	9.78^b^	9.34^c^	0.13	<0.05	<0.05	0.14
Total VFA, mmol/L	72.31^b^	73.33^ab^	76.01^a^	76.94^a^	1.41	<0.05	<0.05	0.97
Mol/100 mol								
Acetate(A)	64.25	63.13	63.63	64.66	1.05	0.48	0.60	0.15
Propionate(P)	18.84^b^	19.44^b^	19.88^ab^	21.47^a^	0.77	<0.05	<0.05	0.36
Butyrate	9.82	9.73	10.69	10.11	0.52	0.25	0.27	0.49
A:P	3.45	3.30	3.29	3.13	0.15	0.19	0.05	0.98

1 LGY, MGY and HGY  =  glycerol-enriched yeast culture at 100, 200 and 300 mL per goat per day, respectively.

abc Means within a row with different superscripts differ (*P*<0.05).

## Discussion

It has been demonstrated that some yeast strains can produce and accumulate one or more compatible solutes intracellularly under a hyperosmotic environment to counteract intracellular water flow into the medium. For example, glycerol is the most prominent compatible solute in *S. cerevisiae*, *Candida glycerinogenes*, *Candida krusei* and so on [21]–[23]. Based on the physiological characteristics of yeast cell, glycerol is generally produced by fermentation under hyperosmotic environment with several typical glycerol producing yeasts, such as *Candida glycerinogenes* [22] and *Candida krusei* [23], which can produce about 137 g/L and 47.7 g/L glycerol under the hyperosmotic environment, respectively. However, no glycerol producing yeasts are available for animal diet as direct-fed microbes permitted by the FDA, in spite of high glycerol yield. Therefore, in this study we preferred to isolate an osmotolerant *S. cerevisiae* (a direct-fed microbe permitted by the FDA) from unprocessed honey.

As the most important fermental condition for glycerol synthesis, hyperosmotic environment was frequently achieved with salts (mainly NaCl and sulfite) in previous studies [24]. However, NaCl was used in this study because sulfite was not well for health of animal. Furthermore, *S. cerevisiae* is very tolerant to high concentration of NaCl, 12% (120 g/L) or even higher [25], thus, 30 g/L (3%) NaCl was selected as the optimal concentration in the present study due to the highest glycerol yield and its not seriously affecting the growth of *S. cerevisiae*. Under this hyperosmotic environment and other optimized fermentation conditions, 38.7 g/L glycerol was obtained by fermentation with S. cerevisiae in this study. Perhaps the glycerol yield was a bit low, however, high glycerol yield would be achieved by improving fermental pattern in future researches.

In this study, the final broth of GY, including glycerol and yeast cells, was first proposed to be fed to ruminant. Higher plasma glucose concentration was observed in the goats supplemented with GY, in agreement with the findings of previous studies about feeding glycerol to ewes [6], [26] and cows [7], [27]. This result was probably attributed to the well-known glucogenic effect of glycerol. According to Krehbiel (2008), 43% glycerol is directly absorbed across the ruminal wall and 44% glycerol is converted to propionate, butyrate, and other products by fermentation of ruminal bacteria when glycerol is directly added to the rumen [28]. The absorbed glycerol and ruminal propionate, both as glucose precursors, are converted to glucose via the gluconeogenesis pathway in the liver. Therefore, it is likely that GY supplementation improved the energy status of goats as evidenced by increased plasma glucose, which is of great significance for ruminants, especially peripartal ruminants. Because ruminants in perinatal period frequently enter a state of negative energy balance (the cause of energy metabolic diseases) due to decreased dry matter intake and increased energy demands of lactation. That is also why the study has been conducted. However, other research reported that serum glucose concentration decreases in lambs fed with crude glycerin as a replacement of corn in diets [29]. The inconsistent effect of glycerol on blood glucose can be attributed to the decreased dry matter intake in that study, or the decreasing concentrations of starch contained in that glycerol diets, which affected the rate of passage and resulted in less fermentation in the rumen and more absorption in the small intestine [29]. Goats supplemented with GY have higher plasma total protein concentration than those not supplemented with GY. This result can be attributed to the well-known impact of yeast on rumen fermentation and nutrient digestibility, which enhanced ammonia uptake and improved microbial protein production [30], [31]. In this study, GY supplementation did not affect the concentrations of triglyceride and total cholesterol, suggesting that GY did not increase the burden of lipid metabolism on the liver and thus ensured the health of the ruminant.

In the present study, ruminal propionate production was increased by GY supplementation, similar to the findings of many previous studies on sheep fed with glycerol or yeast [32], [33]. Supplement of GY altered ruminal fermentation pattern from acetate to propionate production, as evidenced by the linear reduction in the ratio of A: P with increasing GY dose. The increase in ruminal propionate (a primary glucose precursor), which might account for a portion of the increase in plasma glucose, was either a result of the conversion of glycerol to propionate by ruminal bacteria [32], or associated with the enhanced fermentation of dietary nonstructural carbohydrates into propionate by amylolytic bacteria stimulated by yeast [33], thus contributed to a reduction in the ratio of A: P. The increase in the total VFA concentration was largely driven by the increase in propionate concentration from 13.6 mM to 16.5 mM (data not shown). This result is consistent with the decrease in ruminal pH. The goats supplemented with GY had lower ruminal NH_3_N content, which is consistent with previous findings that NH_3_N content is reduced in lambs and lactating cows supplemented with yeast additive [11], [31], [34]. Stimulation of the growth of ruminal microbial populations by yeast would increase NH_3_N consumption and improve microbial protein production. Cellulolytic bacteria derive their N exclusively from NH_3_N [35]. Nevertheless, NH_3_N concentration was still above 5 mg/dL, which is said to be necessary to support optimal microbial growth [36].

## Conclusions

In this study, a glycerol-producing yeast strain (*S. cerevisiae*) was successfully isolated from unprocessed honey. GY containing 38.7±0.6 g/L glycerol and 12.6±0.5 g/L biomass was also successfully prepared. Supplying GY to goats increased the plasma glucose concentration, ruminal VFA, and molar proportion of propionate; decreased the NH_3_N; and altered the ruminal fermentation pattern from acetate to propionate production. The results of this study indicated that GY has great potential to be developed as a new feed additive with dual benefits of glycerol and yeast for ruminants.
